# Clinical Examination of the Chest

**Published:** 1910-03-26

**Authors:** S. H. Habershon

**Affiliations:** Physician to the Brompton Hospital


### Hospital Clinics.

### CLINICAL EXAMINATION OF THE CHEST.

By S. H. HABERSHON, M.D., F.R.C.P.; Physician to the Brompton Hospital.

(A Lecture Delivered at the Hospital for Consumption and Diseases of the Chest, Brompton, on January 26, 1910.)

Gentlemen,—I must ask your indulgence this afternoon, because I shall deal with a subject which may at first seem to be very elementary; but there is no harm, I think, in delving at once into the foundation of things and going over some subjects that we may perhaps have long thought were at the very elements of our examination, and therefore were not necessary to enter into sufficiently carefully to be made the subject of a lecture. The tendency of the present day is to minimise the value of careful clinical examination. The use of *x*-rays, the surgical method of exploratory operation, and the most modern means of treating disease by examination of the blood and by vaccines, introduce the great danger of ignoring and depreciating the importance of careful clinical examination and observation. While I am fully alive to the aid to diagnosis which is afforded by these methods, I am equally conscious that these cannot with safety be dissevered from the actual and careful examination of the physical signs. In all diseases of the chest this is sufficiently obvious. The importance, first of all, of a routine examination to be conducted in every case cannot be over-estimated. The practitioner of medicine and the student are advised to commence with routine and to practise it throughout life, and never to neglect it in any single case, whatever may be the complaint of the patient.

Now, in all examinations it is very necessary for the doctor to assume a comfortable position, so that his attention is not distracted in any way. That is especially obvious when the examination is done at the bedside and the doctor has to lean over the patient; particularly so when the floors are polished, as in this Hospital, so that the feet tend to slip away. This latter is the reason I carry two stethoscopes, and why I always advise everyone to do the same. For accuracy of examination the majority of the physicians of this Hospital recommend the single stethoscope. The conduction of sounds, especially those of the lungs, is, to my mind, very much better with the single stethoscope than with the double. And let me call your attention to the best form of the single stethoscope. It is very much better not to have any breach of continuity in it. I do not like single stethoscopes which are made of two different kinds of wood, or which screw together. This one I show you is of ebony and is all in one piece.

It is self-evident that you can best examine a patient when there is no external noise to direct your attention away from the patient.

The next point is as to the position of the patient. It is always preferable when examining the chest that the patient be standing, for difficulties arise when he is lying in bed; respiration is not then so free, and the anterior parts of the lungs expand more than the posterior, so that there is a diminution of the cardiac dulness. I have verified this in patients both in the out-patient room and in the wards. On the other hand, cardiac cases, even if first examined with the patient standing, should always be examined in recumbency, because very often a cardiac bruit is either heard only in the recumbent position or is intensified. This is especially true of the mitral murmur. The patient should be directed to stand on both feet, with the arms loosely at the sides. Standing on one leg alters the position of the scapula, and such muscles as the trapezius, the supraspinatus, etc., and will often produce a slight difference in the percussion note on the two sides. Such difference is observed when there is hip disease —*i.e.* when one leg is shorter than the other. When examining in the recumbent position the patient should be lying on the back. When lying on the side there is a difference in percussion note due to the muscles, owing to pressure.

In *auscultation* of the chest, especially when a respiratory murmur is being examined, it is essential that the patient should not pant or make any noise with the mouth; the breathing must be deep and silent, with the lips slightly parted. I have often seen doctors deceived by the noise made by the patient's mouth; by this means, indeed, bronchial or broncho-cavernous breathing may be simulated. In cases of laryngitis and in that group where there is stertor this cannot be avoided.

Before proceeding to the routine physical examination of the chest, it is of vital importance to be acquainted with all the outlines. In this short space of time I cannot go into the anatomy of the chest, but you should be well acquainted with the outlines of the lungs and their lobes in relation to the skeletal walls of the thorax. I call your attention to the diagrams in use in this hospital, which are some of the best which have ever been employed. Perhaps I ought not to say that as I was commissioned to prepare them. But I may say I took nearly a year to produce them, after visiting many post-mortem rooms and anatomy rooms, and they have been approved by Professor Peter Thompson, who was at that time the Professor of Anatomy in King's College. They are from Professor His' model of the chest, which is found in the Middlesex Hospital. It is the chest of a young adult or child, and therefore it had to be modified a little to get this outline of an adult chest. One of the important points is the inter-lobar septum, between the right upper and middle lobes, which you will find follows precisely the lower border of the fourth rib. Also note that the interlobar septa between the upper and lower lobes behind commence at different levels on the two sides. The right corresponds to the lower border of the lateral spine of the fourth dorsal vertebra, whereas the left interlobar septum corresponds to the upper border of the lateral process on the left side of the fourth dorsal vertebra.

On looking at the bare chest we see the ribs rising and falling with respiration, and the first thing to note is the *expansion* of the chest. Whatever be the shape of the chest, good expansion is an indication of the condition of health and vigour. The horizontal measurement at the nipple level ought to increase on good expansion by two or three inches, this being the difference between forced inspiration and forced expiration. There is no hard and fast line which you can set to mark deficiency, but anything less than one to one and a half inches shows decided deficiency. But even in the perfectly healthy person breathing is not always conducted perfectly. But the depth of the breathing can be increased by experience and by practice. One's attention is first directed to the shape of the chest, and especially the condition in the subclavicular region. Those who have to do with life assurance know that this is one of the most important questions in the report. It is one of the chief features in the chest of a person of a so-called "tuberculous diathesis," or one whom we should more correctly say had inherited a poor physique.

Next we come to *palpation*, and in this we of course, observe anything like abnormal pulsation, and the position of the apex beat, its character, and the vibrations of the voice; also the difference between the vibrations on the two sides. And one would feel for any palpable vibration such as that of pleural friction, or of rhonchus or sibilus.

The examination of the *percussion* note requires a good deal of practice. And let me say it does not depend on any particular note, nor on the *timbre* of that note. Its appreciation is not easier to a person with a musical ear, but depends more on what is known as the muscular sense, the feeling of resistance. Next I want to tell you of a plan which I always adopt in my wards, and recommend to everyone else. You will find you will bring out the resistance of dulness in the chest very much better if you use a wooden door as a resonator. Place your patient against a solid pitch-pine door, or let him lie down on a wooden stage such as we have in this hall; and I believe I shall be able to illustrate this point in some of the patients whom I shall ask you to examine presently, who have very slight physical signs. Let the heels, the back of the head, and as much of the spine as possible touch the door. The very element of percussion consists in keeping the end of the finger pressed quite firmly down on the chest. I prefer to percuss on the first phalangeal joint of any of the fingers. Next remember that you do not need *momentum* for correct and good percussion: do not allow the arm to be raised for the purpose, and only the hand from the wrist. What you want is the piano-player's movement.

I now pass to *auscultation*. And I want here to mention briefly the nomenclature which I use. Ordinary natural breathing is *vesicular*. If the expiration becomes slightly high-pitched, which is of the character we know as bronchial, and which as a rule denotes some consolidation, we call it *broncho-vesicular*. But if the expiration is typically high, we know it as *bronchial* breathing. If the bronchial breathing is of that loud and marked character which you get when there is complete consolidation around the bronchi, so that you hear the sound of air passing through the large bronchi and conducted to the exterior, I prefer to limit to that breathing the term *tubular*. If the breathing, besides being high-pitched, has a larger wave and becomes more hollow, then we use the adjective "*cavernous*." The earliest type I prefer to call "*broncho-cavernous*"—that is to say, half-way between bronchial and cavernous. For breathing in which the wave-length is very large and indicates reverberations of a large air-containing cavity we use the term "*amphoric*" breathing, which introduces with it almost musical reverberation.

Next, especially in early cases, one looks for an indication of superadded sounds which we know as *râles* or *crepitations*. And râles is perhaps a wider term than crepitations, because by the latter we only mean crackling sounds, whereas râles mean any foreign or superadded sound, such as clicking, or creaking, or croaking, or other sounds. There are one or two points which it is important to emphasise with regard to these superadded sounds. They are of a dry or a moist character. I need scarcely describe the common dry sounds, such as those heard in bronchitis, or, at all events, in bronchial catarrh, the snoring and creaking rhonchus and sibilus. Others are associated with pleural friction, and are known to us all. But some of the crackling sounds, which are also dry, are rather more difficult to diagnose. In cases of emphysema we very often hear a few crackling sounds with inspiration only in the supraclavicular region, and these sounds are both variable and fleeting. Those are the râles which Laennec described as *crepitous*. I do not say they are heard only in cases of emphysema, for they are often heard by the first person who goes to listen to a chest. I believe they are due to the opening up of somewhat collapsed air-vesicles, and when you come to examine your patient or put him through a series of pulmonary gymnastics, and make him breathe more deeply than he has been accustomed to for a long time, you will elicit sharp crackling sounds at the apices of the lungs. We must beware of these, because they are often mistaken for crepitant sounds of more serious import. The other dry crepitant sounds are connected with old pleurisy, especially with adhesions. They are more often of a very sharp character, and generally with inspiration. They are known as *adhesion râles*, are caused by the stretching and creaking of small pleural adhesions due to old pleurisy, and often heard in the site of an old pleuro-pneumonia. Sometimes they are very difficult to distinguish from moist sounds, and I have more than once been deceived, and only been enlightened by the patient telling me that I need not pay attention to any crackling sounds at the base of the right lung as they had been there for 20 years. These crepitant sounds are medium in size. There are a very few dry sounds, somewhat larger, which are heard at the edge of advancing tubercle. The dry sounds known as fine-hair crepitations are due to intense congestion of lung, and they are heard in the early stage of pneumonia, even before red hepatisation has set in, and are probably due to the opening up of vesicles which have been partially collapsed by the engorgement of the vessels. When caused at the edge of advancing tubercle they are larger than are the dry râles of tubercle and pneumonia, and we call them *sub-crepitant* because they are smaller than the ordinary crepitant râles. The crepitations or moist râles which we hear in early tuberculosis vary very much in size. As a rule, if they are not of the sub-crepitant kind they are medium in size, but they vary much in their dependence on the respiratory effort. And there is one point which I always teach, and which I believe makes an immense difference in the interest we take in our patients who are suffering from tuberculosis, and on which I lay very great stress because of its importance in diagnosis and prognosis. I contend that if a doctor is examining a patient with tuberculosis who has crepitant râles, he ought, when blindfolded, to be able to tell us at once whether the case is one of active and acute and progressive breaking down, or whether the lung is gradually becoming drier, or whether the disease is becoming quiescent, and whether it is almost quiescent. The method is very simple, and depends entirely on the relation of moist sounds to inspiration and expiration. I have never published these observations, but I think they are worth recording. I will ask you to verify them for yourself on the patients whom I have brought for you to examine, and also in your own practice.

When air bubbles through an exceedingly liquid secretion you would naturally expect to hear a rattling sound to and fro—*i.e.* with inspiration and expiration. When there is acute progressive disease, with active breaking down, the secretion from the lungs is of a more liquid character than in the later stages, when the disease is quieting down, and therefore you always hear, in acute or progressive disease in the earliest stages, the crepitant sounds with inspiration and expiration, *double râles*. I cannot lay too much emphasis on this. I cannot show you many of these patients, because they are in bed. As a rule they are accompanied by acute symptoms, profuse sweating, or by rise of temperature or an irregular temperature. Sometimes you hear them in a patient who is improving. More commonly these crepitant sounds are associated with a widely oscillating temperature, and all the other physical signs of active progressive disease. What happens when the secretion becomes drier, more viscid, and sticky? You would expect that when the person draws in the breath this glutinous expectoration would be thrust aside, and it would adhere to the walls of the smaller bronchioles, making a crackling sound when the air enters; but the way having been already cleared when the patient expires air, there would no longer be crackles. That is the second stage. I call these the *first two degrees* of crepitant sounds. When the secretion is still more viscid you will often hear no crepitant sound at all with ordinary respiration; and this brings me to the importance of the cough as a means of diagnosis. Ask your patient to cough and then to take in a breath, and you will at once hear a shower of crepitant râles. This is the *third stage*, and the most favourable of all—the crepitant râles are on the verge of disappearing. That is heard constantly at Frimley Sanatorium. In bronchitis I have not laid emphasis upon it, as it is not of so much aid in the diagnosis of the case. In the moist stages you will hear crepitations with inspiration and expiration, and very often in later stages, when the secretion is drying up, you may hear the crepitant sounds only with inspiration. But let me ask you to use your own observation in this matter. You will find it of value in your diagnosis of the condition and the prognosis also, and it will enable you to note the weekly progress of your patient. If a house physician comes to me and says "I think this patient is better; there are fewer râles than the last time," I say "How do you know?" It is merely a matter of opinion; there are no means by which you can accurately remember. But it is a very different thing if you examine a patient, say, on admission to hospital, and find crepitations on inspiration and expiration, and in a month's time you find that the expiratory crepitations have disappeared, and they are heard only on inspiration. You can then emphatically say that the process is one of amelioration, and that will be confirmed by a glance at the temperature chart.

By *auscultatory percussion* you will be able to recognise the margin between the edge of the heart and a large pleural effusion; you will be able to know where fluid ends and the solid heart begins. You will be able to define the margin of a mediastinal tumour, and whether dulness is caused by one large tumour or by a consolidation of the sternal margin of the opposing lungs, such as we get sometimes in turberculous disease.

The common method which my colleague, Dr. Maguire, and myself use is by the finger tap. By that means one knows the probable position of the septum. You must remember that the vibrations caused by the finger tap are conducted not only through the thoracic walls, but through the deep structures, and that therefore there is a breach of continuity, such as I spoke of, which is caused by the dipping down of the two layers of pleura to form the interlobar septum. You will find that while you are tapping upon the lobe over which your stethoscope is placed the vibrations which you will hear will be loud and distinct, but directly you pass over the margin they are damped and they sound distant. Exactly the same happens where the cardiac dulness is lost in that of a pleural effusion. By ordinary percussion you cannot tell any line of difference. But by auscultatory percussion you will find that directly your finger tap passes over the line between the cardiac dulness of the edge of the fluid the sound is damped. I prefer to use a tuning-fork. The regions where it is very easy to detect the lines of interlobar septa are, first of all, the lines between the upper and lower lobes in the axilla. It is more difficult in a woman, because the mammary gland masks the physical sign. The lines of the interlobar septa can also be easily detected in the region between the scapula and the vertebræ behind; but not over the scapula, because the bony resonance interferes.

